# No Time for Waste! Co‐Producing Practice Guidelines and Research Recommendations to Promote Earlier Diagnosis of Colorectal Cancer in Community Pharmacies: A Qualitative Analysis

**DOI:** 10.1111/hex.70411

**Published:** 2025-09-12

**Authors:** Evelyn Brunsdon, Shakira Hollyfield, Sanjana Challa Venkat, Aradhna Kaushal, Willie Hamilton, Lindy Berkman, Stephen Rowley, Adam Todd, Andy Husband, Christian von Wagner

**Affiliations:** ^1^ Institute of Epidemiology and Healthcare University College London London UK; ^2^ King's College London London UK; ^3^ School of Pharmacy Newcastle University Newcastle UK; ^4^ University of Exeter Exeter UK; ^5^ Co‐Applicant and PPI Lead Public Co‐Applicant UK; ^6^ PPI Representative Public Co‐Applicant UK; ^7^ Newcastle NIHR Patient Safety Research Collaborative (PSRC) Newcastle UK

**Keywords:** access, bowel cancer, co‐production, colorectal cancer, community pharmacy, early diagnosis, Nominal Group Technique

## Abstract

**Introduction:**

In the United Kingdom, community pharmacists (CPs) are increasingly being used to provide supplemental care for patients with specific conditions while the NHS is under pressure from declining GP numbers alongside a rising demand for services. CPs are ideally placed to contribute to early diagnosis of colorectal cancer (CRC) through extended services, especially amongst deprived areas where access is higher across all deprivation deciles in urban areas. However, they need to be embedded within integrated pathways with clear lines of communication and co‐operation between CPs and other healthcare professionals. There is not yet a clear understanding of the challenges and barriers to this integration, or the best way forward to expand CP services to include earlier CRC diagnosis. The present study aimed to co‐produce a set of practice guidelines and research recommendations about how community pharmacy can facilitate early CRC diagnosis.

**Methods:**

We ran a series of three co‐production workshops across two sites (six total) in London and Newcastle (2023–2024). Nominal Group Technique was chosen to structure each co‐production session and provided the basis of our workshop guide. Workshops were audio recorded and transcribed for qualitative analysis using both a framework approach and an inductive content analysis, which is the focus of this paper.

**Results:**

The salient issues CP staff face include assessing individual risk, incorporating additional services into existing workloads and financial constraints, advertising these services effectively, better use of physical space to allay CP users' privacy concerns, and finding ways better with other healthcare providers.

**Conclusion:**

Expanding CP services to include screening efforts for CRC is achievable in the short term through practical actions. Key recommendations include addressing privacy concerns for pharmacy customers when discussing CRC symptoms, better utilising and expanding digital communication tools to facilitate closer working relationships between CPs and other healthcare professionals, and providing adequate incentives, screening and support materials to CPs, including FIT kits.

**Patient or Public Contribution:**

PPIE input was central throughout all stages of this study including study conception via our public co‐applicant (L. B.), methodology and study conduct. We held meetings of both a patient advisory group and a steering committee of academics and PPIE representatives throughout the project. Our steering committee held two meetings, once at the onset of study development, and again prior to our final dissemination workshop. This consisted of nine people, one of whom was a patient representative. Our patient advisory group met twice during our project, comprised of ten people, five of whom were patient representatives along with five members of the research team. This group met to review our process after completion of the first workshop at each site, and then again after completion of all workshops to plan our final consolidation meeting. Each workshop was also facilitated by our public co‐applicant and, in some cases, other patient representatives, who met regularly with other facilitators during the organising and running of each workshop. Members of our PPIE group contributed to the paper write up as co‐authors and led on the creation of study dissemination materials, including a video podcast and website.

## 1 Introduction

In the United Kingdom, community pharmacists are increasingly being seen as a potential alternative or augmentation to GP appointments for certain conditions [1]. With the NHS Community Pharmacist Consultation Service, which has run since 2019, and the Pharmacy First services launched in 2024 [2], more patients are able to access healthcare services sooner by utilising their local pharmacy [3]. This comes at a time when the NHS is under significant pressure from declining GP numbers alongside a rising demand for services [3], and the NHS Bowel Cancer Screening Programme, introduced in 2006, has continued to expand eligibility, firstly in 2010, and again in 2021 to lower the eligibility age to 50 [4].

CPs are in an ideal position to contribute to early diagnosis of colorectal cancer (CRC) through these extended services, especially amongst more deprived areas where access is higher across all deprivation deciles in urban areas when compared to GP premises [5]. Every day about 1.6 million people visit a pharmacy in England [6], many of whom already receive help with short term relief from symptoms that may be indicative of cancer. A recent analysis of CRUK Health Professionals Tracker survey of CP staff showed that 8 out of 10 respondents reported that they occasionally encouraged patients to spot or respond to cancer signs and symptoms [7]. However, very few did so regularly. CPs already play an important role in illness prevention.

A Cochrane Review of CP interventions included 58 randomised controlled trials focusing on diabetes, hypertension, asthma and modification of cardiovascular risk, including approaches to screening and prevention [8]. The potential for CPs to support the early diagnosis of cancer has been recognised more recently [9, 10] with a review of 12 papers assessing the role of CP in delivering cancer diagnosis initiatives [9]. Other research suggests there is scope for CPs to use patient purchasing behaviour to target CRC awareness activities. A qualitative study of patients diagnosed with lung, CRC or gastro‐oesophageal cancer in the preceding 12 months reported that symptoms were often managed by lifestyle changes and purchasing medicines from local shops, supermarkets and pharmacies [10].

A systematic review aiming to understand which aspects of pharmacist‐led medication reviews were associated with positive outcomes, such as adherence to medication regimens and reduced number of hospital re‐admissions, identified the lack of connectivity, both physical and digital, between CP based interventions and other primary care services as a key challenge [11].

Some of these challenges relate to practical implementation of services, which can strain the relationship between primary care and CPs. Another systematic review examined pharmacists' and GPs' views of pharmacy services extending beyond traditional medicines supply functions [12], which highlighted how access to medical data and lack of adequate remuneration act as barriers to implementation. Other challenges include the selective conditions most appropriate for therapeutic review by pharmacists, and effectively advertising this information. For example, the public still lack awareness of the skills of pharmacy staff [13], which may impede the uptake of new services. The acceptability of extended services is also likely to be affected by the set‐up and space within pharmacies [14]. There is evidence of CPs providing successful public health services where discretion is necessary, which may be of relevance when discussing CRC symptoms with patients [8]. Other examples of CP extended services where discretion is required include chlamydia testing [15].

In Australia, 21 community pharmacies recruited 91 consecutive users seeking advice for CRC symptoms or who were asking to purchase products used to treat diarrhoea, constipation or haemorrhoids [16, 17]. Pharmacy users were asked to complete a Patient Consultation Questionnaire [18], which led to eight primary care referrals, of which five were attended. In the UK, the Royal Pharmaceutical Society collected data on people reporting to the pharmacy with CRC symptoms, and how staff responded [19, 20]. A total of 498 community pharmacists took part in the survey. Around 70% of sites saw at least one user reporting with CRC symptoms within the 2 week data collection period. In all cases, pharmacists gave oral and written information. An analysis of GP referrals showed that pharmacists were able to distinguish between high‐ and low‐risk symptoms. Furthermore, Badenhorst et al. [21] assessed the frequency of symptoms suggestive of cancer (alarm symptoms) across 33 community pharmacies over 6 months in the North of England. During the study period, 642 people presented with alarm symptoms, including potential CRC warning signs, such as rectal bleeding, persistent diarrhoea and unexplained weight loss.

As the nature of CRC screening in CPs would differ from these examples, prior to implementing any changes the experiences and perspectives of CP staff must be included. These challenges to expanding CP services are a lack of training about CRC awareness, and a lack of cohesive and integrated actions for community pharmacists to support early diagnosis. Capturing CP staff perspectives poses an opportunity for knowledge exchange and the development of a clear roadmap for better integrating expanded services.

By co‐designing a series of workshops directly engaging key stakeholder groups, such as CP staff, we are able to not only create this guidance but ensure it is grounded in the direct experience of the intended audience.

### 1.1 Aims and Objectives

Primary aim:

- To co‐produce a set of practice guidelines and research recommendations about how community pharmacies can facilitate early CRC diagnosis.

Specific objectives:

- To elicit and rank ideas on if and how community pharmacy staff could (i) promote awareness of screening and symptoms, (ii) undertake individual CRC risk assessment and (iii) make integrated referrals.
- To incorporate variations in attitudes (as expressed during workshops) towards community pharmacy supporting early diagnosis of CRC by sex, ethnicity, professional background and region.

This paper is reporting on a qualitative analysis conducted within the project.

## 2 Methods

**Figure 1:**
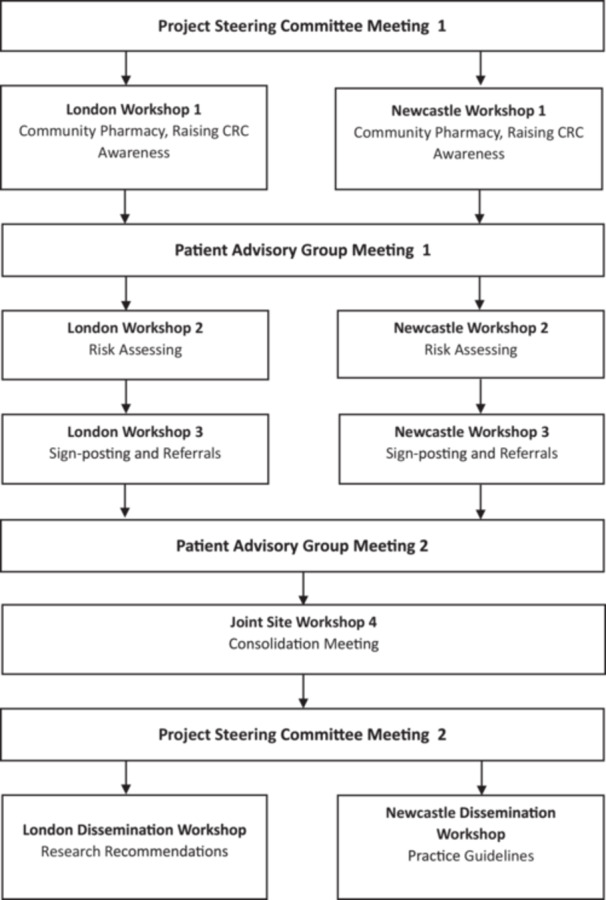
Project stages alongside Patient Advisory Group and Project Steering Committee contributions.

We ran three co‐production workshops across two sites in London and Newcastle (six in total) between 2023 and 2024 (see Figure 1). These two areas were chosen to reflect how regional differences in CRC incidence, geography, socioeconomic status and ethnic background could affect individuals' views about the role of CP in the early diagnosis of cancer [16]. All sessions were facilitated by members of the research team, namely the Co‐PIs, PPIE lead, and research assistants. A consensus‐generating participatory methodology called Nominal Group Technique (NGT) was chosen to structure each co‐production session and provided the basis of our workshop guides [22, 23, 24] and consisted of four steps:

(1) Silent generation of ideas, (2) Round Robin, (3) Assembly/plenary discussion and (4) Voting and ranking. This paper focuses on the qualitative analysis of transcripts (predominantly of Steps 2 and 3). Further details can be found in our NIHR final report [25].

### 2.1 Participants

Recruitment for the London workshop was facilitated by the Clinical Research Network, Middlesex Group of Local Pharmaceutical Committees, and Taylor MacKenzie market research services. Participants for the Newcastle workshops were recruited through Taylor McKenzie market research services and snowballing amongst staff at a School of Pharmacy, Newcastle University. In total, there were 31 participants across all the workshops in Newcastle with some individuals participating in multiple sessions. There were 16 members of the public, 7 community pharmacists who worked in a pharmacy setting and 7 GPs. We also had a number of academics attend the Newcastle meetings all of whom had interests in community pharmacy and in aspects of the proposed methods.

In London, a total of 51 participants were recruited. 47 attended the co‐production workshops and 4 pharmacists participated in our supplementary one‐to‐one interviews, which we offered to prospective participants who could not attend a workshop or preferred a one‐to‐one interview for other reasons. A total of 14 were primary healthcare professionals, 16 were community pharmacists or worked in a pharmacy setting, 17 were members of the public, and 4 were expert witnesses, such as screening practitioners, health promotion specialists, cancer epidemiologists, and gastroenterologists.

### 2.2 Workshop 1: Awareness Raising

Workshop 1 focused on awareness raising of CRC in CP. After a brief introduction to CRC, As part of NGT, participants generated ideas by themselves (Silent generation of ideas), and shared them with the group (Round Robin). During the Round Robin, members of the research team (including the two patient representatives) started generating themes (Assembly) before reflecting them back to the group as a series of recommendations (Plenary discussions). Finally, participants were asked to rank the recommendations by assigning 1 vote (*least important*) to 5 votes (*most important*) to each recommendation (Voting).

### 2.3 Workshop 2: Risk Assessing

Workshop 2 followed the same approach as outlined for Workshop 1 to co‐produce ideas to the question ‘*What can community pharmacies do to identify people at above average risk of CRC?’* This included an introduction to risk assessment tools (RATs [25]) informed by a co‐investigator. Members of the public were invited to discuss their experience of attending a CP with symptom presentation and CP staff were asked the frequency with which, in their experience, people attend the pharmacy to discuss symptoms.

### 2.4 Workshop 3: Signposting

Workshop 3 put CP back into the broader context of other health providers. The facilitator asked the group to consider ‘*What can CP staff do to sign‐post people of higher CRC risk to relevant services*?’. Participants were provided with an overview of the findings from Workshops 1–2, followed by a brief introduction to the use of home‐based stool testing as part of the NHS Bowel Cancer Screening Programme and more recently to triage people with low‐risk versus high‐risk symptoms.

### 2.5 Analysis

Data analysis from NGT and reporting of results were carried out using a combination of qualitative and quantitative methods. Data were recorded on flip charts during the meeting and supported by audio‐recording for subsequent transcription. Quantitative analysis from the scoring and ranking was used to identify priorities. Participants rated the importance of the top 10 items from 10 (*most important*) to 1 (*least important*). Each participant awarded 100 points to the most important item, with the remaining items being given a score between 99 and 0. The single most important item received the most points.

We used a blended approach to analyse qualitative data through an iterative process of on‐going coding review amongst members of the research team (E. B., S. H., S. C. V). We began by using a framework analysis [26] to code transcripts with NVivo14 software into the identified areas of importance for policy changes and research recommendations (e.g., improving collaboration between health professionals) as decided over the course of our workshops. After coding, a spot‐check was conducted using a frequency analysis to check whether the amount of data items coded into the framework matched with the ranked order of importance of themes. From this analysis, we extracted quotes to better understand individual and group thinking. Subsequently, we used a deductive approach based on the COM‐B behaviour change model [27] as a theoretical framework to map the views of CP staff, in terms of their perceived capability, opportunity and motivation to engage with increasing CRC screening awareness and risk assessment. The present results section is structured according to the higher order themes as derived from the initial framework analysis. Within these framework items, we further stratified data into ‘child‐nodes’, which reflected the differing topic areas relevant to each framework item. These child‐nodes followed an inductive content analysis (ICA) [28] approach, wherein the groupings were created from the different topics within the data itself.

## 3 Results

### 3.1 Pharmacist Role, Environment and Autonomy

Across workshops participants discussed acceptability of the pharmacist as a credible source of cancer‐related information, what role the pharmacist can or should play in cancer screening, and whether community pharmacy would offer enough privacy when discussing potentially embarrassing CRC symptoms within the CP. It was felt that, without doing so, this hesitancy would put people off from using the CP to seek treatment and access further testing.

‘If you're going to get patients coming in or patients coming into a community pharmacy, I think British people generally, are very embarrassed talking about poo. …I think people struggle to go and see their doctor about it, and I think that they could perceive it would be potentially even more embarrassing a scenario, to talk to a pharmacist about it perhaps, because of the lack of privacy’.

B11

Suggestions included the ways the pharmacy environment could be adapted to better allow for privacy. Private consultation rooms exist in some pharmacies, though many people, especially those who have not typically seen the pharmacy as an alternative to their GP, may not be aware of this. Placing more visible signs within the pharmacy waiting area, as well as giving pharmacy users ways of requesting a meeting in the private consultation rooms were suggested. This could include both paper slips left on the counter, or adding options into electronic interfaces used for patient check‐ins at primary care services.

‘You mustn't alienate someone and embarrass them because if it's crowded and there's a load of people queuing, they don't want to be kind of saying that, because they might then feel embarrassed and not come back to you… It defeats the object really’.

A16

Another notable point was that a sense of familiarity with the pharmacy staff may help alleviate patient anxieties and allow for more open conversations. This allows for the pharmacist or counter staff to ask further questions about specific symptoms more informally.

‘I thought about when someone is presenting to buy a product for diarrhoea or constipation then you could offer, the counter staff could offer a follow phone call to the patient if symptoms don't go away after a set amount of time. So, a bit like the new medicine service where when you start a new medicine we ring you in a week's time to see how you're getting on. We could do the same thing again but the pharmacist rings just to say, have things cleared up or has it got worse’.

B27

Interestingly, it was noted that the familiarity with pharmacy staff in rural areas could instead be a hindrance to raising embarrassing symptoms.

‘The pharmacy that I work in, it's in a really small rural village, everyone knows everyone. So if you have a patient come to the counter more than likely it'll be their neighbour who is the counter assistant or whatever, so actually maybe that feeds into a comment made earlier about people potentially prefer to speak to their pharmacists because generally the pharmacist doesn't live in the village’.

B2X

Participants also discussed how the change in role of the pharmacist to provide more clinical services, such as CRC screening, could be aligned with the revenue generation priorities of pharmacies. While it was recognised that pharmacists are contractors like GPs, the influence from a corporate environment within larger chains was seen as a central concern.

‘I think in a beautiful world, we sit down and we do what we've been trained to do best…. but we know that money makes the world go round… [pharmacists] go into community pharmacy and within a year they absolutely hate it… They had so much passion to help people but… it's just how to get these targets… I know these people want to spend time with these patients [and]guarantee they get the best care, but they don't have the complete autonomy to decide that on their own. They're working under a branch manager who's working under a regional manager, who's working under a superintendent, who at the end of the day, at the end of the month, it's these numbers that they discuss’

(1. REF 27 PAR(M)).

Addressing this would require recognising the competing priorities for pharmacist time as well as the incentivisation structures for pharmacies participating in other public health programmes, such as additional funding for flu vaccinations.

Within this expanded role, participants felt more needed to be done to allow CPs to follow up if the symptoms persisted and to enable pharmacy autonomy to make referrals and order tests.

### 3.2 Raising Awareness and Public Health Campaigns

Participants felt CPs could spearhead awareness campaigns by advertising the common symptoms of CRC within the pharmacy itself, with posters and signs at the counter or on TV screens in waiting areas, and normalising conversations about bowel cancer.

Participants felt it was important that messaging strategies consider the potential anxiety that comes with discussing cancer in relation to current symptoms.

I think the first thing would be to get somebody to say, ‘I've got a problem, I need to sort it’ and ask the pharmacist, ‘I think I've got one or two of those symptoms, what should I do?’

B15

To help with this, a focus on prevention and the benefits to outcomes when diagnosed early was stressed as an important part of any awareness campaign. Participants noted the importance of currently employed means for public health messaging, such as the use of posters and leaflets with translations into other languages common within different communities, or social media promotional campaigns, but felt this could be expanded on within the CP. This included putting leaflets within prescription bags, advertising the ability to have a consultation with the pharmacist, or co‐ordinating further with the local Integrated Care Boards (ICBs).

### 3.3 Improving Communication and Collaboration Between Healthcare Professionals

Participants discussed the importance of further integrating pharmacy staff and services with broader healthcare providers and improving communication with GPs and hospitals. This included informal meetings and closer working relationships between nearby GPs and pharmacies and expanding on existing digital tools, such as PharmOutcomes or Sonar, to help with communication and include the pharmacy in service provision. Pharmacies not yet using PharmOutcomes should be encouraged to train their staff with the platform and more broadly for this software to be better utilised.

‘PharmOutcomes and Sonar are what community pharmacists are using, for example when we do the flu vaccinations we will do them and that's how the GP surgeries are notified. We're doing the blood pressure, testing in community pharmacies, some pharmacies are having to do it manually where they're sent emails. Some pharmacies have it set up on their systems where we can record [referrals] and it's just directly sent to the GP surgeries, so we don't have to worry about emailing it. So like the colleague just said, that it has to be adapted, so, yes, there could be things added on to it where a reminder pops up to say, could the pharmacists contact the patient or check or send a text to see if it's been done or if it's linked. If I've sent a referral to the centre, if the centre then sends a message back that we haven't heard from the patient or something in a week or ten days, so there are steps that can be added’.

A35

Participants noted pharmacies near to GPs or hospitals should do more to introduce and familiarise staff to allow for more informal working relationships. These connections could create more opportunities for patient referral, discussion of symptoms, and feedback to the pharmacy following referrals. Participants also discussed whether making both nearby GPs and the ICBs aware of the pharmacy's participation in more active screening and patient engagement for CRC would allow for a co‐ordinated effort to improve management of red flag symptoms. As one GP described:

‘The pharmacy was in our building, and we were very friendly with them, we knew them. On more than one occasion, I've had our pharmacist say to us, “I had a lady in, she was wanting to buy Imodium, but actually, on close questioning, she's been losing weight, she's had a change in bowel habits… would you mind seeing her?” Yes, I'll squeeze her in, just tell her to take a seat etc. and I'll add her to my list. I genuinely think the fact that we had a really good working relationship with them, and they felt they could approach us and they knew their customers well enough… on more than one occasion, I have certainly seen people after a request from the pharmacist, because with two or three they've got alarm bell symptom, alarm bell symptom, alarm bell symptom’.

B12

### 3.4 Targeting Those at Risk and Providing Kits for People With Symptoms

Much of the discussion around how to identify people at risk focused on using purchasing data to identify individuals repeatedly buying medications, however participants found this topic of the discussion to be relatively divisive and opinions were mixed. Concerns were raised about a customer's right to privacy as well as whether contacting individuals about their purchase history would deter that individual from returning to the pharmacy. Participants agreed that while there is a need for pharmacies to be more proactive in recognising red‐flag symptoms a balance was required.

‘I personally don't like anyone contacting me to say actually we've found out that you like this product, do you want to talk to me about it? No I'll call you if I need to, you know, so that's really I find that really annoying so if it was me I'd go into a pharmacy that asks a question that was very specific and not be called upon to say you've bought X amount of packets of paracetamol last month, have you got a repeat headache you know. No, I don't want anyone doing that’.

B22

After identifying an individual at risk or having conversations with a customer about their symptoms, participants highlighted the need to have a clear plan for what comes next. RATs were seen as having far more potential benefit here than was currently being used. Participants felt providing a clear percentage chance for increased potential risk based on different symptoms would help CPs in decision making while also providing the patient more accessible information to share with their GP. Specifically, participants frequently identified the opportunity here to supply Faecal Immunochemical test kit:

‘I think supplying FIT tests to the eligible patient on request, seems like a very natural thing’.

B114

### 3.5 Support, Training, Education for Pharmacists

Participants recognised that expanding pharmacy services would need to be accompanied by further investment in support and training for pharmacy staff. An area of concern was the workload of pharmacists and the competing demands for their time and resources which CRC screening would require. There was a recognition that while an increase in funding and staff availability, such as including a healthcare assistant within the pharmacy, would be a more effective solution, in practice these constraints are unlikely to be overcome in the immediate term. Discussion instead focused on creating and making freely available training materials and courses for staff and preregistration pharmacists to assist with the pharmacists' responsibilities, take over tasks where appropriate, and to streamline the screening process with easy‐to‐use check lists or flowcharts. It was felt counter staff could benefit from further education on CRC red‐flag symptoms, and pharmacists from the creation of CRC symptom tools to refer patients more quickly and effectively.

‘So as a pharmacist…. That's sometimes the opinion. I thought just upskill the rest of the team, create more accuracy checking technicians so that pharmacists can provide a more patient centred role’.

B2X

Participants agreed that many questions raised on how to best support and provide training materials would require targeted research. These areas of research would need to include the performance metrics pharmacists face and how to incorporate these into changes to their current services, how to incentivise a pharmacy without financial support, and how training materials can be best incorporated into existing routes for pharmacy staff development. This study should maintain the distinction between chain pharmacies and smaller independent pharmacies as the potential constraints and pressures they face may differ.

‘Often especially if you're in the big like corporate chains… we are told you check 200 scripts a day or you do something else that makes us money otherwise patient doesn't matter’.

B2X

### 3.6 Improving Inclusion and Accessibility for Underserved Communities

Participants recognised the barrier that charging for test kits would have on individuals experiencing red flag symptoms, particularly those with financial difficulties. Promoting a paid for test kit following a discussion about the risk of CRC with a customer could potentially undermine trust and be detrimental to improving inclusion for underserved communities.

‘I think that's your biggest block. So the pharmacist says to you, and you've lost weight, you think you're losing weight, you might have bowel cancer, here, you can purchase a kit from me. That, to me, is commercialised. It feels disingenuous. So I think the public health message is lost there for me completely’.

A128

Within these communities, participants raised concerns about the impact of the COVID‐19 pandemic leaving people disillusioned with the healthcare system. It was felt these considerations would need to form a part of any public health messaging or screening initiative, but would require further dedicated research. Practical steps participants raised included ensuring language translations were made readily available, and considerations made for sharing information without relying overly on digital forms of communication. Highlighted for further research were the ways that cultural norms across different groups could act as a barrier to discussing CRC symptoms, or how previous negative experiences with the healthcare system impacts on perceptions and use of pharmacy services.

## 4 Discussion

Our findings can help to facilitate a practical roll out of expanded community pharmacy services in CRC screening and identify areas for future research. The salient issues CPs face include: targeting individuals at risk, incorporating additional services into existing workloads and financial constraints, advertising these services effectively, better use of physical space and considerations of customer privacy concerns, and finding ways to work closer in better collaboration with other healthcare providers.

Previous research within this area suggests that access to medical data between GPs and pharmacy services, as well as inadequate remuneration, act as barriers to extended pharmacy services beyond supplying medicines [12]. Both of these issues were similarly identified within our analysis. Our study added specific value by providing new insights into how this could be implemented. For example, suggestions were made to expand the existing use of digital tools of communication between community pharmacies and nearby GPs, such as but not limited to Sonar or PharmOutcomes, to allow for notes and alarm bell symptoms to be flagged and shared. Further research is needed here to understand the interoperability of these systems, how they are currently being used, and whether other bespoke communication software packages would be more effective at improving means to follow up with a GP after referring patients experiencing alarm bell symptoms.

The second issue of adequate remuneration was seen as a necessary but difficult component of expanding CP roles. Participants recognised that pharmacy staff, while good intentioned and with the desire to provide better and more helpful services to patients, could be restrained by funding availability and, in some instances, a larger financial incentive structure of chain pharmacies generating revenue. Aligning these competing priorities with the goal of expanded CRC screening was seen as the next priority for future research.

Previous research identified that acceptability of extended services within CPs can be affected by the physical set‐up and space within pharmacies, specifically the ability to provide discretion when necessary [14]. This mirrors another salient topic within our analysis. Our findings suggest there are existing practical options available to CPs, which could help alleviate this issue without inordinate time and expense. For instance, participants suggested the existing presence of private consultation rooms within CP could be better utilised, and the ways in which individuals request to use these be expanded to allow for more subtle requests. Specific proposals included physical request cards available in waiting areas which can be handed into CP reception staff, the inclusion of additional options on interactive electronic check‐in screens (where used) in collaborating primary care services, or both physical and digital materials within waiting areas advertising the expanded services and availability of private consultations.

Our findings suggest that a future priority for research is how to best identify those at risk of CRC within the CP setting. We identified numerous important topic areas within this. First, multiple questions are raised around individual privacy. Whether or not patient purchasing data can or should be used to target individuals for screening was divisive and will require careful consideration. In current studies, anxiety around data misuse is raised as a barrier to acceptability [29]. Another area for future research our findings point towards is how CP staff best communicate and target their advertising of expanded services in a way that is sensitive to anxieties which may arise while discussing possible cancer symptoms. Similarly, efforts will need to be made to better understand the types of tools which best assist CP staff in identifying at risk patients, such as RATs or CRC symptom tools, how they are used, and how best to incorporate support and training materials into existing CP workloads and incentive structures. Our PPIE group felt it was important to highlight the significance of the age of the population with CRC, as people who fall below the current screening age of 50 may be overlooked, and agreed these considerations should form part of future research plans into how CP staff can best identify individuals at risk.

Our work also highlights the need to better understand how the COVID‐19 pandemic has shifted people's perception of CPs and the acceptability of expanding their role to include other services such as cancer screening. Of particular importance here was how previous experiences amongst already underserved and underrepresented communities informs willingness and desire to engage with additional CP services. Messaging and advertisement campaigns would also need to take this into account, as would training and materials provided to pharmacy staff to guide conversations with customers about their symptoms and the options for screening. We found that providing FIT kits within CPs was considered by participants to be beneficial, but would need to accompany these others steps to ensure their effective use and safety netting by CP.

*Research recommendations*: There is the potential for larger pieces of work to continue on from this project to try and produce a clear referral pathway for a patient who presents at the community pharmacy with alarm symptoms. At present there are many informal routes or local arrangements to support this. Our work clearly outlines these routes should be formalised and made more reliable for the patient and the health professionals involved. The same argument applies to the use of screening kits and how we can use community pharmacies to increase uptake of screening. Our participants outlined the requirement to have reliable systems of referral or the issuing of screening kits to be embedded in existing services, and not standalone approaches, which may have the potential to increase workload and cause confusion for the public. Further work could define the best approach to this system, and has the potential in the long‐term to examine how much impact such services could have on earlier CRC diagnosis. Specific areas for future research identified by this study have been listed on the findings page of our study website, phabric.org.uk.

### 4.1 Limitations and Strengths

The audio quality of group workshop recordings led to gaps in discussion within the transcripts. The study was constrained by the length of time available for each workshop, where full discussion amongst larger groups of people is limited for everyone to have an opportunity to speak. Another limitation affects the generalisability of our results, as the level of representation for our stakeholder groups was inconsistent across the London and Newcastle cohorts. By allowing repeat participation of some group members across different workshops, some opinions may have a larger presence within our data.

A strength of this study was the diverse range of views gathered through a multi‐site selection and our co‐production approach with an integrated PPIE group. We chose NGT to elicit responses from each group member to both pre‐determined structured questions, to generate data about specific topics, and use group discussion to prioritise. The NGT approach facilitates knowledge and idea exchange on issues of shared interest to identify areas of consensus and establish priorities for change. Importantly, for the aims of this project, the collaborative nature of NGT has been found to increase stakeholders' ownership of the ensuing research and therefore raises the likelihood of changing clinical practice and policy [30, 31, 32]. By using anonymous list generation, we hoped to avoid undue influence between groups and the impact of dominant voices, especially those seen as expert.

## 5 Conclusion

Expanding CP services to include screening efforts for CRC is achievable in the short term through practical actions. Key recommendations include addressing privacy concerns for pharmacy customers when discussing CRC symptoms, better utilising and expanding digital communication tools to facilitate closer working relationships between CPs and other healthcare professionals, and providing adequate incentives, screening and support materials to CPs, including FIT kits. We identified future areas of research which could assist in the effectiveness of these services, including how to message and advertise expanded CP services, how to communicate risk with pharmacy customers, and to identify and target at‐risk individuals for screening. A full list of research areas can be found on our study website phabric.org.uk.

## Author Contributions

**Evelyn Brunsdon:** formal analysis, data curation, supervision, writing – original draft, writing – review and editing, investigation. **Shakira Hollyfield:** project administration, investigation, data curation, formal analysis, writing – review and editing. **Sanjana Challa Venkat:** investigation, formal analysis, writing – review and editing, project administration. **Aradhna Kaushal:** writing – review and editing. **Willie Hamilton:** conceptualisation, investigation, writing – review and editing. **Lindy Berkman:** funding acquisition, investigation, writing – review and editing. **Stephen Rowley:** writing – review and editing. **Adam Todd:** funding acquisition, investigation, supervision, writing – review and editing. **Andy Husband:** conceptualisation, funding acquisition, supervision, formal analysis, writing – review and editing. **Christian Wagner:** conceptualisation, funding acquisition, supervision, formal analysis, writing – review and editing.

## Ethics Statement

Ethical approval was sought from UCL Research Ethics Committee and granted in 2022 Ethics Application 1024/006: Co‐producing practice guidelines and research recommendations to support early diagnosis of colorectal cancer in community pharmacy.

## Conflicts of Interest

The authors declare no conflicts of interest.

## Data Availability

The data that support the findings of this study are available on request from the corresponding author. The data are not publicly available due to privacy or ethical restrictions.

## References

[1] “Pharmacy First: What You Need to Know,” Department of Health and Social Care Media Centre, accessed October 14, 2024, https://healthmedia.blog.gov.uk/2024/02/01/pharmacy-first-what-you-need-to-know/.

[2] “Pharmacy First,” NHS England, accessed October 14, 2024, https://www.england.nhs.uk/primary-care/pharmacy/pharmacy-services/pharmacy-first/.

[3] “Pressures in General Practice Data Analysis,” accessed October 14, 2024, https://www.bma.org.uk/advice-and-support/nhs-delivery-and-workforce/pressures/pressures-in-general-practice-data-analysis.

[4] “Bowel Cancer Screening Annual Report 2021 to 2022,” GOV.UK, accessed October 25, 2024, https://www.gov.uk/government/publications/bowel-cancer-screening-annual-report-2021-to-2022/bowel-cancer-screening-annual-report-2021-to-2022.

[5] A. Todd , A. Copeland , A. Husband , A. Kasim , and C. Bambra , “Access All Areas? An Area‐Level Analysis of Accessibility to General Practice and Community Pharmacy Services in England by Urbanity and Social Deprivation,” BMJ Open 5, no. 5 (2015): e007328, 10.1136/BMJOPEN-2014-007328. PMC4431167 25956762

[6] “Essential Facts, Stats and Quotes Relating to Pharmacy and Pharmacy Professionals,” accessed July 3, 2021, https://psnc.org.uk/services-commissioning/essential-facts-stats-and-quotes-relating-to-pharmacy-and-pharmacy-professionals/.

[7] R. S. Kerrison , A. Robinson , H. Skrobanski , et al., “Demographic and Psychological Predictors of Community Pharmacists' Cancer‐Related Conversations With Patients: A Cross‐Sectional Analysis and Survey Study,” BMC Health Services Research 22, no. 1 (2022): 1–11, 10.1186/S12913-022-07587-1/TABLES/5. 35227265 PMC8883634

[8] L. Steed , R. Sohanpal , A. Todd , et al., “Community Pharmacy Interventions for Health Promotion: Effects on Professional Practice and Health Outcomes,” Cochrane Database of Systematic Reviews 12, no. 12 (2019): 011207, 10.1002/14651858.CD011207.PUB2. PMC6896091 31808563

[9] L. Lindsey , A. Husband , H. Nazar , and A. Todd , “Promoting the Early Detection of Cancer: A Systematic Review of Community Pharmacy‐Based Education and Screening Interventions,” Cancer Epidemiology 39, no. 5 (2015): 673–681, 10.1016/J.CANEP.2015.07.011. 26272518

[10] F. Notman , T. Porteous , P. Murchie , and C. M. Bond , “Do Pharmacists Contribute to Patients' Management of Symptoms Suggestive of Cancer: A Qualitative Study,” International Journal of Pharmacy Practice 27, no. 2 (2019): 131–139, 10.1111/IJPP.12489. 30251300

[11] A. Husband and A. Robinson‐Barella , “Integration and Connection: The Key to Effectiveness of Large‐Scale Pharmacist‐Led Medication Reviews?,” BMJ Quality & Safety 33 (2024): 765–768, 10.1136/BMJQS-2024-017740. 39214679

[12] A. M. K. Hindi , S. Jacobs , and E. I. Schafheutle , “Solidarity or Dissonance? A Systematic Review of Pharmacist and GP Views on Community Pharmacy Services in the UK,” Health & Social Care in the Community 27, no. 3 (2019): 565–598, 10.1111/HSC.12618. 30047617

[13] A. M. K. Hindi , E. I. Schafheutle , and S. Jacobs , “Patient and Public Perspectives of Community Pharmacies in the United Kingdom: A Systematic Review,” Health Expectations 21, no. 2 (2018): 409–428, 10.1111/HEX.12639. 29114971 PMC5867331

[14] A. M. K. Hindi , E. I. Schafheutle , and S. Jacobs , “Community Pharmacy Integration Within the Primary Care Pathway for People With Long‐Term Conditions: A Focus Group Study of Patients', Pharmacists' and GPs' Experiences and Expectations,” BMC Family Practice 20, no. 1 (2019): 26, 10.1186/S12875-019-0912-0/FIGURES/2. 30736732 PMC6368723

[15] P. Baraitser , V. Pearce , J. Holmes , N. Horne , and P. M. Boynton , “Chlamydia Testing in Community Pharmacies: Evaluation of a Feasibility Pilot in South East London,” Quality and Safety in Health Care 16, no. 4 (2007): 303–307, 10.1136/QSHC.2006.020883. 17693680 PMC2464947

[16] M. Jiwa , S. Sargant , J. Hughes , et al., “Triaging Consumers Who Present Bowel Cancer Symptoms to Community Pharmacies: A Pilot of Two Interventions,” Australian Pharmacist 28 (2009): 348–351.

[17] M. Jiwa , D. Sriram , Z. Khadaroo , and W. C. Ping‐Delfos , “Could Community Pharmacies Offer an Opportunity to Improve Outcomes for Patients With Bowel Cancer?,” Quality in Primary Care 19 (2001): 105–108. 21575332

[18] S. Selvachandran , R. Hodder , M. Ballal , P. Jones , and D. Cade , “Prediction of Colorectal Cancer by a Patient Consultation Questionnaire and Scoring System: A Prospective Study,” Lancet 360 (2002): 278–283. 12147370 10.1016/s0140-6736(02)09549-1

[19] E. Kennington , S. Carter , M. Dear , B. Allen , and N. Patel , “From Abstracts of the Royal Pharmaceutical Society (RPS) Annual Conference 2012, 9–10 September 2012, Birmingham, UK,” supplement, International Journal of Pharmacy Practice, 20, no.S2 (2012): 19–20.

[20] Royal Pharmaceutical Society, “Utilising Community Pharmacists to Support People With Cancer,” (2020), file:///D:/uitlising%20community%20pharmacists%20to%20suport%20people%20with%20cancer.pdf.

[21] J. Badenhorst , A. Todd , L. Lindsey , J. Ling , and A. Husband , “Widening the Scope for Early Cancer Detection: Identification of Alarm Symptoms by Community Pharmacies,” International Journal of Clinical Pharmacy 37, no. 3 (June 2015): 465–470, 10.1007/s11096-015-0078-3. 25690464

[22] S. S. McMillan , M. King , and M. P. Tully , “How to Use the Nominal Group and Delphi Techniques,” International Journal of Clinical Pharmacy 38, no. 3 (2016): 655–662, 10.1007/S11096-016-0257-X/FIGURES/2. 26846316 PMC4909789

[23] C. von Wagner , A. Husband , A. Todd , L. Berkman , and W. Hamilton . “Research for Patient Benefit Final Report Form,” National Institute of Health Research, (2024), https://phabric.org.uk/wp-content/uploads/2024/09/NIHR203526_Final-Report2.pdf.

[24] N. Harvey and C. A. Holmes , “Nominal Group Technique: An Effective Method for Obtaining Group Consensus,” International Journal of Nursing Practice 18, no. 2 (2012): 188–194, 10.1111/J.1440-172X.2012.02017.X. 22435983

[25] S. A. Stapley , G. P. Rubin , D. Alsina , E. A. Shephard , M. D. Rutter , and W. T. Hamilton , “Clinical Features of Bowel Disease in Patients Aged < 50 Years in Primary Care: A Large Case‐Control Study,” British Journal of General Practice 67, no. 658 (2017): e336–e344, 10.3399/BJGP17X690425. PMC5409433 28347985

[26] J. Ritchie and L. Spencer , “Qualitative Data Analysis for Applied Policy Research,” in *Analyzing Qualitative Data*, ed. A. Bryman and B. Burgess, 1st ed. (Routledge, 1994), 173–194.

[27] R. West and S. Michie , A Brief Introduction to the COM‐B Model of Behaviour and the PRIME Theory of Motivation [v1] (Qeios, 2020), 10.32388/WW04E6.

[28] M. Q. Patton , Qualitative Evaluation and Research Methods, 2nd Ed. (Sage Publications, Inc, 1990).

[29] Y. Hirst , S. T. Stoffel , H. R. Brewer , L. Timotijevic , M. M. Raats , and J. M. Flanagan , “Understanding Public Attitudes and Willingness to Share Commercial Data for Health Research: Survey Study in the United Kingdom,” JMIR Public Health and Surveillance 9 (2023): e40814, 10.2196/40814. 36951929 PMC10131900

[30] J. M. Bartunek and J. K. Murninghan , “The Nominal Group Technique: Expanding the Basic Procedure and Underlying Assumptions,” Group & Organization Studies 9, no. 3 (1984): 417–432, 10.1177/105960118400900307.

[31] K. Vella , “Use of Consensus Development to Establish National Research Priorities in Critical Care,” BMJ 320, no. 7240 (2000): 976–980, 10.1136/BMJ.320.7240.976. 10753149 PMC27337

[32] “The Nominal Group Technique: A Useful Consensus Methodology in Physiotherapy Research,” Free Online Library, (2014), https://www.thefreelibrary.com/The+nominal+group+technique%3a+a+useful+consensus+methodology+in…-a0160542945.

